# Intermodulation from Unisensory to Multisensory Perception: A Review

**DOI:** 10.3390/brainsci12121617

**Published:** 2022-11-25

**Authors:** Shen Xu, Xiaolin Zhou, Lihan Chen

**Affiliations:** 1 Beijing Key Laboratory of Behavior and Mental Health, School of Psychological and Cognitive Sciences, Peking University, Beijing 100871, China; 2 School of Psychology and Cognitive Science, East China Normal University, Shanghai 200062, China; 3 PKU/McGovern Institute of Brain Research, Peking University, Beijing 100871, China; 4 National Engineering Laboratory for Big Data Analysis and Applications, Peking University, Beijing 100871, China

**Keywords:** frequency-tagging, intermodulation components (IMs), computational model, neural interactions, MVPA, neural network, electroencephalogram (EEG), multisensory

## Abstract

Previous intermodulation (IM) studies have employed two (or more) temporal modulations of a stimulus, with different local elements of the stimulus being modulated by different frequencies. Brain activities of IM obtained mainly from electroencephalograms (EEG) have been analyzed in the frequency domain. As a powerful tool, IM, which can provide a direct and objective physiological measure of neural interaction, has emerged as a promising method to decipher neural interactions in visual perception, and reveal the underlying different perceptual processing levels. In this review, we summarize the recent applications of IM in visual perception, detail the protocols and types of IM, and extend its utility and potential applications to the multisensory domain. We propose that using IM could prevail in partially revealing the potential hierarchical processing of multisensory information and contribute to a deeper understanding of the underlying brain dynamics.

## 1. Introduction

At every moment, the human brain receives a deluge of information from the world; the source/content of the information may not only be unisensory—visual image, auditory clicks, tactile taps and so on, but also a combination of various inputs from multiple senses. The human sensory system is so powerful that it not only flexibly responds and processes information unimodally or crossmodally, but also integrates local information (such as ingredient properties of an object—colors, temporal frequencies and so on) into a coherent and complete whole. The cognitive processing of the latter has been termed ‘object perception’. Therefore, within ‘object perception’, two processes are on-going potentially: the multisensory perception of an object and the perceptual organization of local information into a global one. For the latter, take the example of the visual (unisensory) system, in which human perception of a figure made of straight lines mobilizes cascades of selective perceptual organizations as well as hierarchical structures of representations [1]. Among the representations, some subsets of a figure will be encoded as integral, structure units of that figure while others will not. The presumed, low-level structural units have been organized to form a holistic perception of the figure of interest.

To better understand the cognitive processing in the above object perception, whether integrating parts into a whole or from unisensory to multisensory perception, we need to further understand the core of the problem: how does the brain dynamically integrate the scattered, local features (representations) into a coherent perceptual object (holistic representation)?

With the rapid development of noninvasive brain technology in the past half-century, our knowledge of unisensory and multisensory perceptual integration has also rapidly advanced. Among various emerging technologies, functional magnetic resonance imaging (fMRI) technology, which captures blood oxygenation level (BOLD)-dependent changes in the human brain, has been a promising tool to address a large domain of neuroscience for human cognition. Most object perception studies with fMRI as the main approach can answer well the following two questions: (1) what are the brain areas that subserve a given typical perceptual integration—this is from the perspective of structural and functional origins for perception; and (2) to explain how perceptual integration has been realized with the functional connectivity among those brain areas identified. However, even with the results answering these two main types of questions, it is still far from clear to probe the exact neural mechanism for unisensory and multisensory processing. Typically, the BOLD signal from fMRI is sluggish to delineate the temporal dynamics of perceptual processing and fails to capture the instant changes in (micro) states of brain function. Moreover, fMRI methods cannot comprehensively reveal the time and frequency-related information that is associated with specific “units” that contribute to the ultimate object perception.

Compared with fMRI, electroencephalogram (EEG) methods prevail due to their high temporal resolutions. However, with traditional EEG approaches, for a multisensory integration exploration, it is difficult to tease apart the contributions from unisensory inputs or the genuine integration of multiple senses. Likewise, it is not sufficient to reveal how the local, elementary components and their interactions lead to the perception of a Gestalt object. To name a typical study, Delis et al. [2] used EEG and an active sensing paradigm in which participants were actively perceiving to distinguish two texture stimuli using either visual (V) or tactile (H) information or two sensory cues (VH) to reveal that the multisensory integration of visual and tactile motor information occurs in the contralateral somatosensory and motor areas. They found that the integration of visual information and tactile information could enhance the accuracy of perceptual decision-making more than unisensory information alone. This study to a certain extent answers the neural mechanism of multisensory integration for perceptual decision-making. However, the readers are not informed how the activated cortices are corresponding to a single sensory input (V or H) or a combination of inputs (V and H) with differential contributions of each sense, but the downside is that we cannot be sure whether/how multisensory integration is occurring in the corresponding brain regions.

Therefore, in both unisensory and multisensory domains, we urgently need to find direct neural markers that can accurately lock and quantify the featured “units” for sensory processing and characterize the integration of the local information into a whole, holistic percept, or integration of the multiple senses into a coherent multisensory representation. This attempt is typically worthwhile when the local information (“units”) could be identified as brain oscillations with characteristic frequency modulations.

In this review, we mainly introduce the intermodulation components (IMs) obtained from steady-state evoked potentials (SSEP) based on frequency-tagging methodologies, in which frequency acts as a feature of stimulus. In previous studies on IMs, researchers have usually designed several stimulus materials that change simultaneously and periodically over time for unisensory information (such as the brightness of stimuli varying sinusoidally in vision research). As observers process this information, their neural oscillations coincide with the input stimuli in terms of the physically eliciting frequencies (cycles), a phenomenon known as “neural entrainment”. Neural entrainment can remain unchanged during information processing, resulting in homeostatic neural responses. Frequency-tagging of neural signals, with frequency as a feature of stimulus in the time domain, could contain power peaks at specific frequencies in the frequency domain. Notably, these frequency peaks generated from external stimuli should be distinguished from the spontaneously generated frequency bands in our brains.

**Figure 1:**
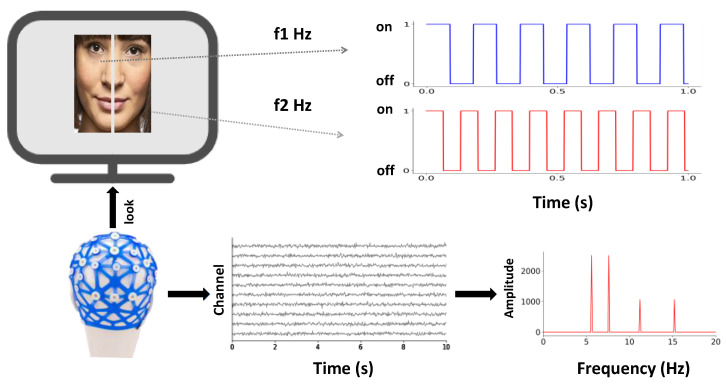
**Principles of the application of IMs in perceptual integration research**. In the study of IMs, the stimulus material generally contains two objects that change with time. Generally, these changing pictures can be periodic changes in brightness (two sine wave signals) or switching changes (two rectangular wave signals). When the subjects were watching these external signals, various signals of specific frequencies were generated in the brain in the form of neural oscillations. These signals contain not only the same periodic signal (fundamental frequency signal) as the external signal does, but also the interaction signal (harmonic signal and modulation signal) generated by the interaction of the signals. By analyzing the EEG signal in the frequency domain, all components of the signal can be obtained. Therefore, the IM approach is a more direct and effective tool for measuring neural interaction at present.

IMs have been frequently used in vision research over the past few decades. The design of experimental materials is the key point of this type of research. Often, the experimental stimuli contain several frequencies (see Figure 1 for f1, f2), and the participants complete the corresponding cognitive tasks. Meanwhile, EEG signals are recorded and then analyzed in the frequency domain. Notably, the power spectrum of the signal has peaks not only at the fundamental frequency (f1, f2), but also at the harmonics of the fundamental frequency (n × f1, n = 1, 2, 3, 4, ……, ∞). For a particular frequency, there are theoretically an infinite number of harmonics, the power of which decreases as its order increases. Furthermore, IMs are also found due to the interaction of the neural signals (m × f1 + n × f2, such as f1 + f2, 2f1 + 2f2, etc.) with the frequency tagging method. Thus, compared to other neuroimaging methods, IMs can provide direct evidence of the types as well as the strengths of unisensory and multisensory integration. Although IMs are often used to study unisensory visual cognition, in recent years, unisensory auditory and tactile stimulation techniques have also been increasingly exploited to study object perception. For example, Flaten et al. [3], using EEG IMs, found that infants were able to integrate top-down musical beats in the early 7–8 months, and the effect was stronger in children whose parents were musicians. Pang et al. [4] used IMs based on steady-state tactile evoked potentials to study the spatial attention of touch; they found that when two closer fingers on the same hand received vibration signals with two corresponding, specific frequencies, EEG results showed that neural interaction can generate intermodulation components (IMs). This indicates that the tactile perception of multiple vibrations on digits could be well accounted for by the underlying IMs, further revealing the role of topological distances between digits in modulating the perceived ‘averaged’ percept of vibrations. Furthermore, extending from this line of research, it is possible to apply IMs to multisensory domains in which each sense could bear its characteristic IM.

In recent years, the IM is no longer limited to addressing the problem of unisensory perception; it is increasingly used to study multisensory perception. For example, Nozaradan et al. [5] explored the integration of visual and auditory information by manipulating the temporal coherence of the stimuli in the adjacent time window. They found that the strength of the integration of visual and auditory information increased significantly when the timing was aligned. Temporal coherence enhanced the processing of multisensory inputs during sensory-specific phases of cortical processing, presumably by inducing synchronous dynamic binding of activity and/or improved dynamic engagement. Porcu et al. [6] manipulated the cues in the experiment (visual cues or tactile cues) and then asked participants to receive 7.5 Hz visual stimuli and 20 Hz tactile stimuli. The results of the study found significant differences in the second-order IM (see Section 2.3) intermodulation responses generated by cues from the two senses. This result suggests differences in the neural integration of tactile and visual information.

In this review, we summarize the recent literature that applied IMs to EEG data to study multisensory perception, detail the principles, the order, and computation modeling of IM, and extend its application in the multisensory domain to better understand the hierarchical perception processing of multisensory information, as well as to reveal the underlying brain dynamics.

## 2. Definitions of IMs

### 2.1. Time and Frequency Domains

Compared with other organisms, humans have highly developed nervous systems and neuron networks, allowing advanced perceptual and cognitive functions. The EEG method can capture the timing signals of the tiny neuronal firings associated with specific cognitive functions. The firings as time series signals may contain various information components and rhythmic information. Therefore, how to separate these rhythmic components is crucial to our understanding of the external environment. The Fourier transform can convert the temporal signal into frequency domain signals to help us separate and identify different information components.

Fourier transform can convert the temporal signal into the frequency domain signal to help us separate different information components. Fourier transform considers that any periodic signal function can be represented by a linear superposition of a set of sine and cosine signals (see Equation (1)).

(1) $$f\left(x\right)=\frac{a_{0}}{2}+\overset{\infty }{\underset{n=1}{\sum }}a_{n}cos\frac{2\pi nx}{T}+b_{n}sin\frac{2\pi nx}{T}$$

where,

$$a_{n}=\frac{2}{T}\int_{x_{0}}^{x_{0}+T}f\left(x\right)cos\frac{2\pi nx}{T}dx,\;b_{n}=\frac{2}{T}\int_{x_{0}}^{x_{0}+T}f\left(x\right)sin\frac{2\pi nx}{T}dx.$$

The above mathematical approach bridges the time domain (wave amplitude varies with time) and the frequency domain (amplitude varies with frequency). Three frequency components, fundamental frequency (e.g., f1, f2), harmonics (e.g., 2 × f1), and IMs (e.g., f1 + f2, which can be represented as m × f1 + n × f2), can be obtained by a frequency domain analysis of time series EEG signals.

Among all Fourier transform algorithms, the fast Fourier transform (FFT) algorithm is one of the most used and is highly efficient. According to the Nyquist theorem, the highest frequency after Fourier transform is half of the sampling frequency in the time domain. Therefore, the experimenter needs to take this into account when collecting EEG signals. In addition, prolonged stimulus presentation can improve frequency resolution (see Equation (2)).

(2) $$r=\frac{P_{m}}{N_{m}}$$

**Figure 2:**
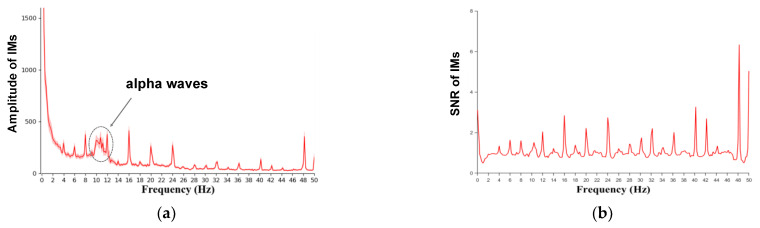
**Amplitude spectrum and signal-to-noise ratios**. (**a**) The alpha wave can be clearly observed, which may overlap with IMs in the spectrogram of the EEG signal. (**b**) The alpha wave is attenuated in the signal-to-noise ratio of the EEG signal and the IM components become clearer than before.

After Fourier transform, Equation (2) r represents the difference between the frequencies of two adjacent data points, $P_{m}$ represents the highest frequency and $N_{m}$ represents the number of data points. According to the Nyquist theorem, it can be obtained that

$$\{\begin{array}{c} P_{m}=\frac{P_{t}}{2}\\ N_{m}=\frac{N}{2}=\frac{T*P_{t}}{2} \end{array}$$

where $P_{t}$ represents the sampling frequency of the EEG signal, N represents the number of data points of the EEG signal and T represents the duration of the stimulus. Thus, Equation (1) can be simplified as r=1/T in which the frequency resolution can be improved with increasing T. In general, we can obtain a spectrogram of the EEG signal under Fourier transform. However, some spontaneous frequency bands (such as alpha waves (8~12 Hz), theta waves (4~7 Hz), etc.) that are independent of external stimuli may overlap with IMs (see Figure 2a). The influence of such frequency bands on IMs can be attenuated by calculating the signal-to-noise ratios (SNR) of the spectral response, as seen in IM studies [7] (see Figure 2b). Hence, the IM method has a high signal-to-noise ratio and is relatively unaffected by artifacts [8]. When calculating the IM response at a given frequency, we considered the peak output at exactly the stimulation frequency relative to the baseline at nearby frequencies [9,10]. For example, the SNR of the spectral response at 10 Hz was computed by dividing the peak output value at 10 Hz by the average amplitude in the range of [9.5, 10.5] Hz (see Equation (3)).

(3) $$SNR_{10\;\mathrm{Hz}}=\frac{P_{10\;\mathrm{Hz}}}{\frac{1}{n}\sum_{i=1}^{n}P_{i}}$$

where $P_{10\;\mathrm{Hz}}$ is the peak output at 10 Hz and $P_{i}$ is the peak output of the i-th nearby frequency in the frequency domain.

### 2.2. Example of IMs

**Figure 3:**
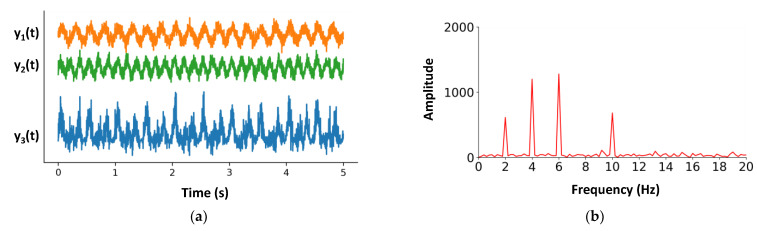
**Simple example of IMs**. (**a**) The three signals varying over time within 5 s. The signals $y_{1}$ and $y_{2}$ are sinusoidal signals with Gaussian random perturbations. The frequencies of $y_{1}$ and $y_{2}$ are 4 Hz and 6 Hz, respectively, and the signal $y_{3}$ is the linear superposition of$\;y_{1}$ and $y_{2}$. (**b**) Amplitude of signal $y_{3}$ in the frequency domain, which represents the amplitude of each spectral component. The term “fundamental” frequencies denote the frequencies of the input signals (4 Hz and 6 Hz here), and the term IM denotes any sum of the nonzero integer-multiples of the fundamental frequencies (i.e., m × f1 + n × f2, such as 4 + 6 = 10 Hz and 6 − 4 = 2 Hz).

To better introduce the principle of IM, we illustrate it with two sinusoidal signals, each having given Gaussian random perturbations:

(4) $$\{\begin{array}{c} y_{1}\left(t\right)=sin\left(2\pi \times 4t\right)+\varepsilon_{1}\\ y_{2}\left(t\right)=sin\left(2\pi \times 6t\right)+\varepsilon_{2} \end{array}$$

where the frequencies of signals $y_{1}$ and $y_{2}$ are $f_{1}=4\;\mathrm{Hz}\;$and $f_{2}=6\;\mathrm{Hz}$. $\varepsilon_{1}\;$and $\varepsilon_{2}$ represent Gaussian random perturbations. The sampling frequency of both signals is designed to be 500 Hz and the duration of the two signals is 5 s. Gaussian random disturbances $\varepsilon_{1}\;$and $\varepsilon_{2}$ are subject to Gaussian distribution N(0,0.5). IMs emerge exclusively from interactions between input signals, so we consider the output signal $y_{3}\left(t\right)=y_{1}\left(t\right)\times y_{2}\left(t\right)+y_{1}\left(t\right)+y_{2}\left(t\right)$ which contains the interaction between the signals, including the two fundamental frequency signals (see Figure 3). Actually, the function used in this example is simpler than that for real neural interaction processes (see Section 3 for details).

### 2.3. The Order of IMs and Its Role in Cognition

The order of IMs represents the sum of the absolute values of the coefficients (such as f1 + f2 is the second-order IM and 2 × f1 + f2 is the third-order IM) in the intermodulation components. For example, in Figure 3, the components at 2 Hz and 10 Hz represent the second-order intermodulation. In task-free vision experiments, the second-order intermodulation response tends to be stronger [11].

**Figure 4:**
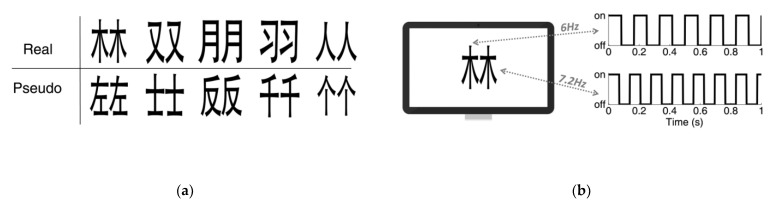
**Stimuli**. (**a**) The real (upper row) and pseudo (lower row) Chinese characters used in the experiment. (**b**) During the experiment, each side of the character was on–off flickering at 6 Hz or 7.2 Hz (balanced across trials).

For example, Cai et al. [12] used the IM-based steady-state visual-evoked potential (SSVEP) technique to study the integration of Chinese characters. In the experiment, participants needed to look at the real characters or fake characters with symmetrical structures flashing on the computer screen. These characters were divided into two independent parts and flashes at different frequencies (6 Hz and 7.2 Hz, see Figure 4). There were no additional tasks for the participants. The results showed significant second-order IM (6 + 7.2 = 13.2 Hz) in both the real characters condition and the fake characters condition. In addition, compared with the fake characters condition, the real characters condition can strengthen the second-order IM (6 + 7.2 = 13.2 Hz) more, with higher peak power than fake characters.

Second-order IMs are considered low-order neural interactions [12]. As this experiment showed, second-order IMs may represent low-level bottom-up cognitive processing, in which participants passively perceived the flickering free from any expectations or focused attention. 

In recent years, studies have found that higher-order IMs may represent neural interactions of higher-order (top-down) cognitive functions (e.g., learning, expectation, attention). Vergeer et al. [11] used EEG frequency tagging technology to guide subjects to complete a figure classification task after receiving four learning sessions to distinguish highly similar, unfamiliar shapes into two categories based on feature combinations. After training, EEG signals were recorded while presenting frequency-tagging shapes from trained or untrained shape families. Post hoc analysis showed a stronger IM response of occipital areas for trained shape families than for untrained shape families. The authors suggest that higher-order IMs may reflect advanced visual computations such as global shape classification.

Several recent studies [13,14,15] have linked the strength of perceptual integration to the order of IMs in their own specific ways. These findings suggest that there may be a relationship between the order of IMs and the level of perceptual processing within the cortical hierarchy [16,17]. Therefore, higher-level neural interactions in the perceptual system may be reflected in higher-order IMs and lower-level neural interactions may be reflected in lower-order IMs. In other words, a cascade of nonlinear operations may lead to the emergence of higher-order or lower-order IMs in corresponding perception processes.

## 3. Modeling for Neural Interaction Processing

There is evidence that IMs are the product of nonlinear perception processing in the brain [18,19,20]. However, how this nonlinear processing is achieved through neural circuits in the brain is unclear. To better understand how IMs are generated and the relationship between input and output, we strongly recommend using computational modeling approaches that play an extremely important role in neural interaction processing to quantify and classify IMs.

To infer the possible neural mechanisms that might cause the observed nonlinear IM responses, Table 1 shows the different mathematical formulae and their respective spectra by the computational modeling approach. Considering the presumed biological plausibility, we focus on three types of nonlinear processing, namely rectification [21], half-squaring [22] and square waves [23,24]. For a sinusoidal input x=sin(2π×f×t), where t is the time and f is the input frequency, we model the rectification nonlinearity as

$$Rec\left(x\right)=\{\begin{array}{c} x,\;(if\;x>0)\\ 0,\;\left(other\right) \end{array}$$

the half-squaring nonlinearity as

$$HSq\left(x\right)=\{\begin{array}{c} x^{2},\;(if\;x>0)\\ 0,\;\left(other\right) \end{array}$$

and the square wave nonlinearity as

$$Sq\left(x\right)=\{\begin{array}{c} 1,\;(if\;x>0)\\ -1,\;\left(other\right) \end{array}$$

Most of the perception processing of the human brain is nonlinear [25,26,27], and not only in one way; processing may be formed by the superposition of multiple nonlinear functions. Therefore, we consider the full model here as the ideal model (see Equation (5)).

**Figure 5:**
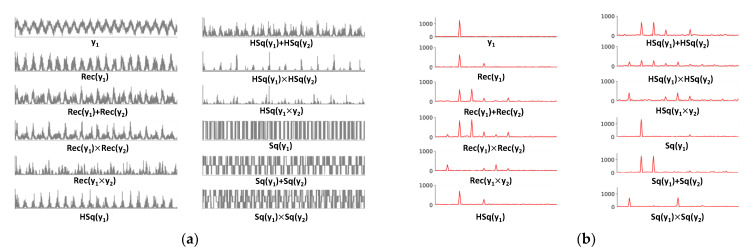
**Examples of different nonlinear processing functions in the time domain and frequency domain**. (**a**) Waveforms in the time domain (0 to 5 s). $y_{1}$ and $y_{2}$ are sinusoidal inputs at 4 Hz and 6 Hz, respectively. See text for definitions of Rec, HSq and Sq. (**b**) Spectra of each waveform in the frequency domain.

These nonlinear functions cannot induce IMs independently except when they are combined with further operations, such as multiplication. Let us check the input and output of different nonlinear models (see Figure 5). Here, we still consider two signals, $y_{1}$ and $y_{2}$, as

$$\{\begin{array}{c} y_{1}\left(t\right)=sin\left(2\pi \times 4t\right)+\varepsilon_{1}\\ y_{2}\left(t\right)=sin\left(2\pi \times 6t\right)+\varepsilon_{2} \end{array}$$

Considering that there are many kinds of nonlinear processing functions, we only consider the Rec function and the Sq function for the sake of simplicity (in fact, the neural interaction may involve more than these two types of functions). First, we use the full model,

$$y=y_{1}+y_{2}+y_{1}\times y_{2}+Rec\left(y_{1}\right)+Rec\left(y_{2}\right)$$

(5) $$+Rec\left(y_{1}\right)\times Rec\left(y_{2}\right)+Rec\left(y_{1}\times y_{2}\right),$$

$$+Sq\left(y_{1}\right)+Sq\left(y_{2}\right)+Sq\left(y_{1}\right)\times Sq\left(y_{2}\right)$$

to perform simulations and generate many simulated EEG signal data. We then fitted the simulated data to the full model (Equation (5)); and generated a second batch of simulated neural data. Correlation analysis was performed on the first set of simulated data and the second set of simulated data. In addition, we need to design multiple reasonable alternative models to verify that our full model is the optimal model (see Table 2).

**Figure 6:**
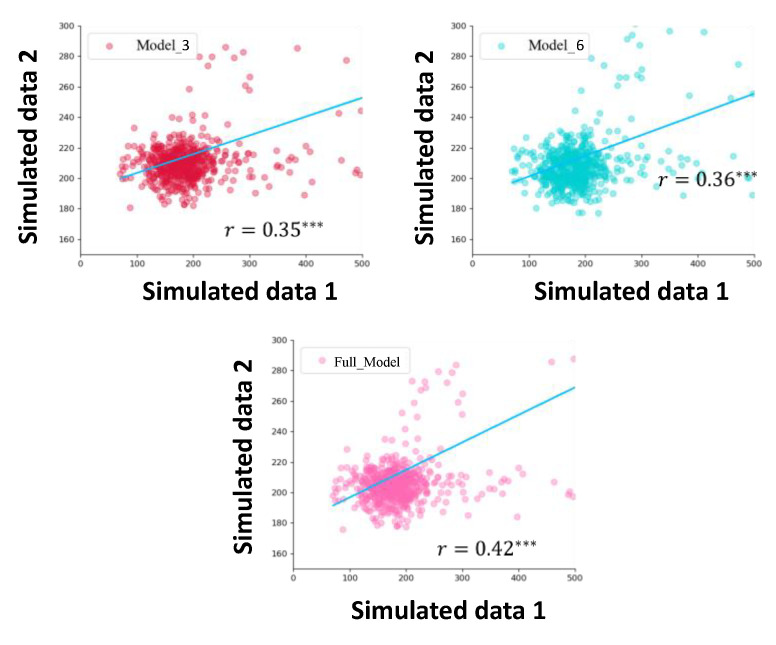
Correlation plot of Simulated data 1 with Simulated data 2 for the full model and two candidate models, where *** represents *p* < 0.001.

To reduce the computer running time, we only compared the full model with Model 3 and Model 6. The posterior predictive check showed that the full model fit better than the other two models (see Figure 6).

**Figure 7:**
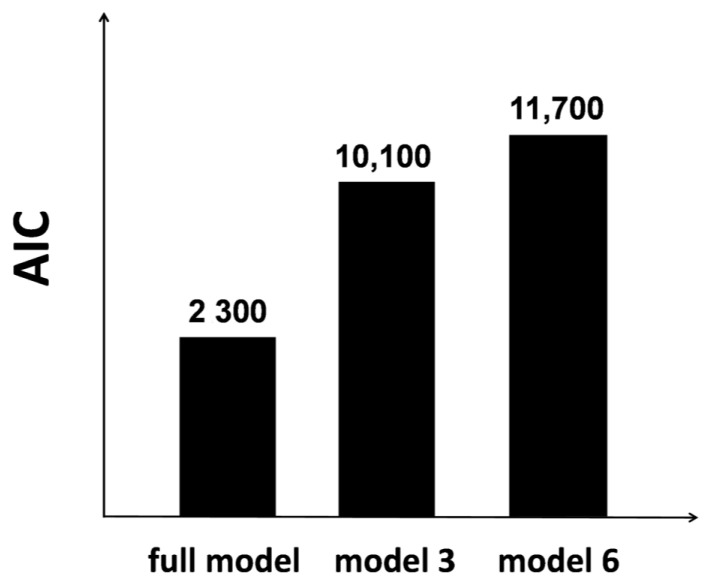
Akaike’s Information Criterion (AIC) for 3 models. The best model is the full model.

In the model fitting step, we used Bayesian estimation to estimate the coefficients of each term in the model. Then, we used the mean in the posterior distribution of the coefficients as a new parameter to simulate the model again. In terms of model comparison, we used Akaike’s Information Criteria (AIC, a smaller value represents a better model) to find that the full model can better fit and predict simulated data (see Figure 7). Therefore, it can be considered that this set of polynomial linear models can explain IMs well.

**Figure 8:**
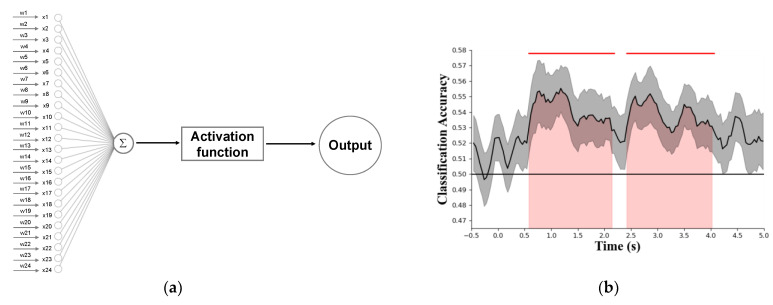
**MVPA algorithm based on single-layer perceptron neural network architecture and decoding accuracy.** (**a**) Input nodes $x_{i}$ denote the SNR or amplitude of 24 EEG channels at the interested IM frequency. $\omega_{i}\;$ denotes the weight of inputs. The sigmoid function is often used as the activation function in the single-layer perceptron neural network. Finally, by summing the weighted signals and nonlinear activation processing, it can be inferred which experimental condition produced the current IM pattern. (**b**) A simulated decoding accuracy profile, time-by-time. The solid black line denotes the chance level of decoding accuracy. The red-shaded area and solid red line above indicates that the single-layer perceptron neural network model can significantly distinguish under which experimental condition the current IM pattern is generated in the current time period.

In addition, the magnitude and pattern of IM responses may differ between different experimental conditions. Traditional statistical techniques (*t*-test, ANOVA, etc.) are sufficient to investigate differences in the magnitude of IMs between conditions. Nevertheless, traditional statistical techniques cannot explain differences in IM patterns between conditions. Multivoxel pattern analysis (MVPA), which is based on the single layer perceptron neural network method (see Figure 8a) has recently been utilized to explore the difference in neuroimage patterns. We consider MVPA as a powerful tool to investigate this type of problem, although to the best of our knowledge, there are no studies yet using MVPA to study the application of IMs in multisensory integration. We can use part of the SNR data of IMs to train this single-layer perceptron neural network model; and another part of the data to test the trained model. When the test accuracy is acceptable, the model can be used to decode new IM patterns, time-by-time (see Figure 8b).

Biological-inspired computational models and model comparison can be compared against the empirical spectrum to gain stronger support for one model than competing models. The above example shows how one can gain insights that go beyond the existence and measurement of neural interactions. Indeed, evidence that provides greater support for a plausible mechanism can be obtained by comparing the results of computational models with empirical data obtained in experiments.

## 4. IMs for Multisensory Perception

### 4.1. IMs Studies of Multisensory Perception

Compared to IMs of unisensory perception, in fact, we can receive information not only from a single sense but also from different senses. Multisensory perceptual processes involve hierarchical brain networks. To better understand this process, Jeffrey et al. [28] provided a great overview of how the targeted manipulation of neural activity using invasive and noninvasive neuromodulation techniques can contribute to our understanding of multisensory processing. In this study, the authors demonstrate that multisensory integration can be a distributed feature across cortical networks and that sensory areas traditionally thought to be dedicated to a single modality can be multimodal. However, before we can do that, we may need more objective neural markers for multisensory integration. In recent years, the principle of IM is no longer limited to the problem of unisensory perception and is increasingly used to study the interaction of multisensory perception.

Typically, the neural maker of multisensory integration has been classically identified in functional magnetic resonance imaging (fMRI) using conjunction analysis. In con-junction analysis, multisensory integration can be inferred if commonly activated (cluster) brain region(s) respond to input from two (or more) sensory modalities. For example, Joassin et al. [29] used fMRI to measure brain activity while participants identified previously learned static faces, voices, and voice-face associations. Using subtraction and conjunction analysis between bimodal and unimodal conditions, they observed that voice–face associations activated visual and auditory regions, as well as specific cross-modal regions located in the left angular gyrus and right hippocampus. Furthermore, functional connectivity analysis confirmed the connectivity of the right hippocampus to the unimodal area. These findings suggest that combined faces and voices rely on brain networks that support different aspects of integration, such as sensory input processing, attention, and memory. Helbig et al. [30] investigated the neural mechanism of integrating visual and tactile shape information through a conjunction analysis of fMRI, and subjects discriminated between bimodal (visual-tactile) or elliptical shapes presented visually alone. A 2 × 5 factorial design manipulated (i) the presence and absence of tactile shape information and (ii) the reliability of visual shape information (five levels). They then investigated whether regional activation of tactile shape discrimination depends on the reliability of visual shape. The results showed that in the primary somatosensory cortex (bilateral BA2) and superior parietal lobes, responses to tactile shape input increased when the reliability of visual shape information decreased. Conversely, tactile input suppressed visual activation in the right posterior fusiform gyrus when visual signals were ambiguous and unreliable. The somatosensory and visual cortex can maintain the integration of visual and tactile shape information through direct connections with visual areas or top-down effects from higher order parietal regions.

However, with its effectiveness in revealing the neural underpinnings, two limitations of conjunction analysis in investigating multisensory interaction currently are that (i) it cannot statistically detect nonlinear interactions of neurons, where signals from one sensory modality modulate responses evoked by the other; (ii) it cannot rule out that the regions obtained by the conjunction analysis are driven by single sensory information. In contrast, the presence of IMs works in a direct fashion, as it clearly shows direct evidence for the existence of nonlinear mechanisms for multisensory interaction. Moreover, in our perspective, the cross-modal interaction contains the interaction of mutual information, which could be described in the framework of IMs as usually being implemented in the visual domain. By deciphering the weighting of each piece of information (source) during multisensory integration, it is promising to unify the causal inference modeling with IM approaches to better account for object perception in the multisensory domain.

### 4.2. IMs in the Spatial and Temporal Rules of Multisensory Perception

For decades, multisensory integration has been understood to take place according to several “rules of thumb” [31,32,33]. Among them, neural interaction responses to multsensory stimuli tend to occur and be enhanced when different sensory stimuli information occurs at the same location (spatial rule) and at the same time (temporal rule). The two rules provide an empirical framework for presumed, successful multisensory integration studies in animals as well as in brain-injured and healthy humans. These two rules may seem simple, but they are often not well satisfied in the real world.

Spatial rules are the result of the superior colliculus, which is known as the area of multisensory integration aligning spatial maps of different sensory information in a similar way. The two rules have been typically summarized in two studies [34,35], realized in the “ventriloquist effect”, which was originally demonstrated in audiovisual interactions, in which the concurrent visual cues could bias the perception of sound location toward the position of the visual distractor (in ‘spatial ventriloquism’), or the sound signal could bias the perceived time onset for the neighboring visual target (‘temporal ventriloquism’), with subjects even trying to ignore them. However, these two rules may seem simple, but they are often not satisfied in reality [34]. Most physical events involve different information from several sensory modalities that occur in different locations and across different points. Compared with traditional imaging techniques, IMs can capture multisensory integration in situations that do not meet the above ‘strict’ yet ‘simple’ spatial rule, or even seemingly violate the ‘spatial’ rule as commonly believed.

**Figure 9:**
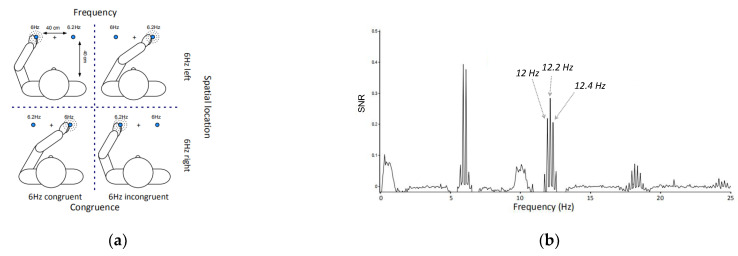
**Experimental design and results.** (**a**) Subjects’ left finger (or right finger) was continuously vibrated at a constant frequency while subjects looked at the continuously flashing light source. The frequencies of tactile and visual stimuli were designed to intersect at 6 Hz and 6.2 Hz. (**b**) Spectra in the frequency domain. The values 12 Hz and 12.4 Hz are second-order harmonics of 6 Hz and 6.2 Hz respectively. Additionally, 12.2 Hz is the second-order intermodulation component (IM).

In a poster demonstration, Keyser et al. [36] utilized the IM method based on a steady-state-evoked potential to study the integration of visual and tactile information. The subjects’ left finger (or right finger) was continuously vibrated at a constant frequency while subjects looked at the continuously flashing light source. The frequencies of tactile and visual stimuli were designed to intersect at 6 Hz and 6.2 Hz (four conditions, see Figure 9). Frequency-domain analysis results revealed an integrated component (6 + 6.2 = 12.2 Hz) of visual and tactile information regardless of the specific spatial locations.

Similarly, Sylvie et al. [5] used the IMs approach, which can characterize the degree and level of multisensory integration to track cortical activity elicited by auditory and visual inputs to explore the effect of temporal coherence on the perceptual combination of multisensory inputs. Their results showed that auditory and visual SSEPs were significantly enhanced in the temporally consistent condition compared to the incongruent condition. In addition, a significant increase in the phase coherence of the two SSEPs was observed. In short, these observations suggest that temporal coherence enhances the processing of multisensory input at sensory-specific stages of cortical processing. With IMs, it is hopeful to pinpoint the detailed, exact time course (as well as temporal dynamics) that subserves the brain oscillations associated with each stage along the multisensory processing hierarchy.

A number of studies have suggested that the IM approach is an effective tool to explore the neural dynamics of multisensory integration in the human brain [37,38,39,40]. The unique ability of IMs provides a direct and objective evidence of neural interactions for both unisensory and multisensory perception, as they are direct measures of these neural interactions.

### 4.3. The Role of IMs in the Relationship between Multisensory Perception and Attention

Multisensory integration is often considered an automatic process in the early stage. Recent studies have shown that multisensory integration can occur at distinct stages of stimulus processing that are associated with and can be modulated by attention. Bottom-up mechanisms can automatically capture attention to multisensory events, whereas top-down attention can facilitate the integration of multisensory inputs and lead to the spread of attention across sensory modalities [41]. 

Recent studies have shown that a multisensory bottom-up process can lead to the capture of attention [42]. From these findings, we can infer that the output of multisensory integration precedes attentional selection, and thus it operates in a preattentional and largely automatic manner. Therefore, for multisensory integration, the bottom-up process may occur early in the overall period, while the top-down process may occur in a later stage, which requires substantial attention. The IM method with MVPA provides an inspiration for exploring the relationship between multisensory integration and attention in the overall period. For example, in a multisensory integration experiment with two conditions, we can use MVPA to fit a single-layer perceptron neural network model, time-by-time, to distinguish between two different experimental conditions over the entire time period. As Figure 8b shows, there is a significant difference between the IMs of the 0.6 s–2.1 s time period and the 2.5 s–4.0 s time period, which means that the MVPA model at these two time periods can distinguish two different conditions. Furthermore, these results demonstrate that multisensory integration is efficiently performed in the participants’ brains. 

**Figure 10:**
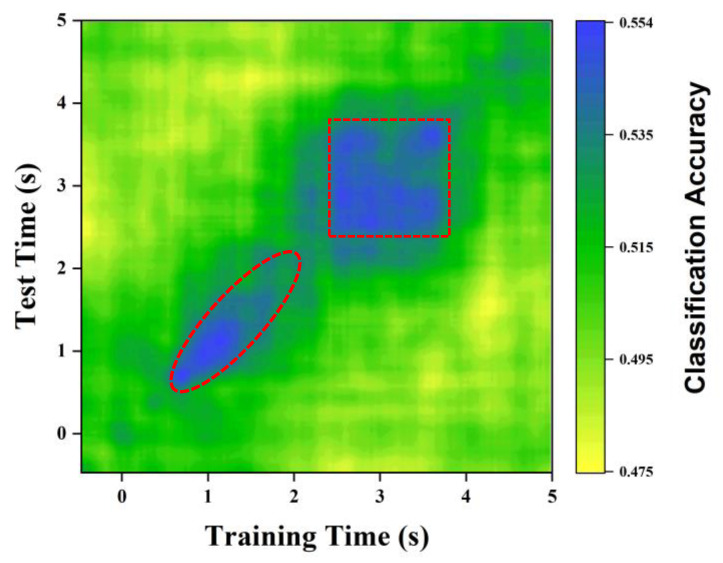
A simulated decoding accuracy profile by cross-temporal MVPA.

However, this time-by-time MVPA approach cannot answer the following question: Is there a difference in the pattern of multisensory integration between these two periods in the brain? We can answer this question with a cross-temporal MVPA approach in which the training dataset and the test dataset are from different periods. This method can help us explore the convergence, which is crucial for exploring the relationship between multisensory perception and attention, of multisensory integration. As Figure 10 shows, there are significant differences in the two time periods of multisensory integration mentioned above. In the time period of 0.6–2.1 s, when the training and test datasets of IMs come from different times, the prediction accuracy of the model is very poor, and the accuracy is large only when the training and test datasets are from the same time (see red diagonal). The two different experimental conditions (training vs. test) produced significantly different IMs, and the encoding pattern of the differences between the two conditions was different from each other time by time. This means that the multisensory integration in the time period of 0.6–2.1 s has not yet converged. This phenomenon of moment-to-moment variation in the encoding pattern of differences between two conditions at an early stage may be due to adaptation of inherent, characteristic differences between the conditions [43,44]. Therefore, we propose that this time period represents the early stage of multisensory integration, which may capture attention (bottom-up) to multisensory stimuli. In the time period of 2.5–4.0 s, the pattern of prediction accuracy is a rectangle (see red rectangle), which means that the prediction accuracy is high regardless of whether the training and test datasets come from the same time span. Therefore, in this period, the encoding pattern for the differences between the two conditions is generalized in a stable fashion. This homogeneity of the encoding pattern of differences at each given time point may suggest that multisensory integration has converged in this period, which may lead to the spread of attention (top-down) [45,46].

In conclusion, the MVPA-based IM approach can provide evidence in exploring the relationship between multisensory integration and attention even though the underlying details for implementation remain open to be further addressed. Additionally, as mentioned in Section 2.3, low-order IMs are involved in low-level cognitive processing and high-order IMs are involved in high-level cognition; it is reasonable to speculate that the theory is also applicable to multisensory integration, even if it is not yet confirmed.

### 4.4. The Role of IMs in the Diagnosis of Pathology by Brain–Computer Interface (BCI)

Brain–computer interface (BCI) systems directly translate signals generated by brain activity into control signals. It is often used in clinical applications and is used for disease diagnosis. Current noninvasive brain signals from BCI include functional magnetic resonance imaging (fMRI), magnetoencephalography (MEG), functional near-infrared spectroscopy (fNIRS) and electroencephalogram (EEG). EEG is portable, reasonably inexpensive with good real-time response, and is technically less demanding than other modalities. That is why it is commonly used in BCI [47]. 

There are significant differences in brain signals between healthy individuals and patients with severe brain injury. Previous EEG-based BCIs have often utilized unisensory IM methods, P300 potential, and event-related desynchronization [48] to perform disease detection. However, these BCI methods are currently facing mainly the problem of a low accuracy of pathological detection. Li et al. [49] introduced a new technique for disease diagnosis, namely multimodal/hybrid BCI, which combines EEG signals with other physiological signals (such as eye gaze, electrocardiography (ECG) or electromyography (EMG)) to solve the above problem.

However, this multimodal BCI method is currently far from being directly applicable to clinical systems because the accuracy of the method is not very stable due to the very large individual differences in patients. We cautiously propose the possibility of combining the multisensory IM method and multimodal BCI for pathological diagnosis, which adds more effective information about the classifier model to the original multimodal BCI method. Therefore, this method may be beneficial to improve the accuracy of pathology detection. In conclusion, this is still an open question.

## 5. Limitations and Conclusions

IM has many advantages but is not without limitations. The limitations of IM as a method for unisensory perception and multisensory perception including (1) this technique is essentially limited to EEG and MEG due to its inherent characteristics, which can lead us to not be able to detect neural activities deep in the brain, (2) the IM method requires that the stimuli in the experimental design must be frequency-dependent. (3) there is no general summary of what cognitive processes correspond to each order of IM components and how to pick the optimal frequency for a given type of sensory stimulus, (4) different cognitive tasks will lead to different types of neuronal interactions, which in turn lead to the emergence of different IMs, but there is not yet clear about how many IMs and which IM to include when analyzing the primary frequency (f1 + f2, 2 × f1 + f2, or 2 × f1 + 2 × f2, etc.), and (5) We all know that IM is the embodiment of neuronal interaction at the macro level. It is not yet known whether we can work backward from IM to the computation of neuronal circuits at the micro level. 

As stated in the introduction, to better understand the neural mechanisms of multisensory perceptual integration, we need to answer not only what regions of the brain represent multisensory perceptual integration but also the degree of multisensory perceptual integration, the type of multisensory perceptual integration and how it happened. This means that we need to deeply and comprehensively understand the spatial, temporal, and frequency rules of multisensory perceptual integration. 

Many previous studies on animal physiology have shown that the processing of perceptual integration is inherently nonlinear. However, most human neuroimaging methods (such as fMRI etc.) relying on “subtraction” to acquire the target neural signals. Due to the inherent linear operations in those methods, they are not suitable for studying such multisensory integration studies that usually involve nonlinear processes [5]. Given that sensory perception processes mostly occur in the sensory cortex, IM-based frequency-tagging has a sufficient spatial resolution to investigate where multisensory integration occurs. In addition, IMs are based on EEG technology and thus have a high temporal resolution to answer the temporal and frequency mechanisms of multisensory integration. Although the order of IMs can be used to explain the hierarchical structure of perceptual integration, in which higher-order IMs represent more higher-level interaction processing and lower-order IMs represent more lower-level interaction processing, empirical implementation has not yet been realized in multisensory domains. This endeavor could be pursued by building mathematics-driven theory with computational simulations and modeling, and by obtaining experimental and empirical data, coupled with advanced analytical methods.

In general, IMs can provide a direct and objective physiological measure of neural interaction not only for unisensory but also for multisensory integration. Both low-level and high-level perceptual processes (e.g., expectation, attention) can be studied using IMs. Although the applications of IMs are less mature for multisensory integration than unisensory integration, its potential value is immeasurable. It holds promise for unraveling the complex hierarchies involved in various interaction processes in human brains.

## Figures and Tables

**Table 1:** Different mathematical formulae and their spectra.

Function	Description	Basis of Neural Processing	Output-IMs	Comment
2$y_{1}$	A single input signal is processed linearly	Neurons transmit signals linearly	Fundamental frequency	Linear processing of signal cannot yield harmonics and IMs
$Rec\left(y_{1}\right)$	Nonlinear half rectification of a single signal	Neuron firing rate is selectively inhibited to 0	Fundamental frequency and 2nd order harmonics	Nonlinear processing of a single signal yields harmonics
$Rec\left(y_{1}\right)+Rec\left(y_{2}\right)$	Two signals are first half rectified and then added	Nonlinear processing of multiple parallel signals	Fundamental frequency and 2nd order harmonics	Nonlinear processing of multiple signals without interaction terms cannot yield IMs
$Rec\left(y_{1}\right)\times Rec\left(y_{2}\right)$	Two signals are first half rectified and then multiplied	Nonlinear Sequence Processing of Multiple Serial Signals	Fundamental frequency, 2nd order harmonics, 2nd order IM and 3rd order IM	Nonlinear processing of multiple signals with interaction terms yields IMs
$HSq\left(y_{1}\right)$	Nonlinear half squaring of a single signal	Neuron firing rate is selectively inhibited to 0	Fundamental frequency	
$HSq\left(y_{1}\right)+HSq\left(y_{2}\right)$	Two signals processed to half squaring nonlinearity and then added	Nonlinear processing of multiple parallel signals	Fundamental frequency and 2nd order harmonics	
$HSq\left(y_{1}\right)\times HSq\left(y_{2}\right)$	Two signals processed to half squaring nonlinearity and multiplied	Nonlinear Sequence Processing of Multiple Serial Signals	Fundamental frequency, 2nd order harmonics and 2nd order IM	
$Sq\left(y_{1}\right)$	Nonlinear square wave of a single signal	Output of ON/OFF neurons	Fundamental frequency, 3rd order harmonics and 5th order harmonics	
$Sq\left(y_{1}\right)+Sq\left(y_{2}\right)$	Two signals processed to squaring wave and then added	Nonlinear processing of multiple parallel signals	Fundamental frequency, 3rd order harmonics and 5th order harmonics	
$Sq\left(y_{1}\right)\times Sq\left(y_{2}\right)$	Two signals processed to squaring wave and then multiplied	Nonlinear Sequence Processing of Multiple Serial Signals	All IMs (low-order and high-order IM)	The interaction of square wave signals can generate many IMs
$e^{y_{1}+y_{2}}/\left(e^{y_{1}+y_{2}}+1\right)$	Sum of the two signals as the input of logistic function	Sum of multiple neuron signals as input for logical selection	Fundamental frequency and 3rd order IM	
$e^{y_{1}-y_{2}}/\left(e^{y_{1}-y_{2}}+1\right)$	Difference of the two signals as the input of logistic function	Difference of neuron signals as input for logical selection	Fundamental frequency and 3rd order IM	

**Table 2:** Candidate models are used for model comparison. a, b and c denote the weights of the models.

Model ID	Model Function
Model 1	$a\times Rec\left(y_{1}\right)+b\times Rec\left(y_{2}\right)$
Model 2	$a\times Rec\left(y_{1}\right)+b\times Rec\left(y_{2}\right)+c\times Rec\left(y_{1}\right)\times Rec\left(y_{2}\right)$
Model 3	$a\times Rec\left(y_{1}\right)+b\times Rec\left(y_{2}\right)+c\times Rec\left(y_{1}\times y_{2}\right)$
Model 4	$a\times Sq\left(y_{1}\right)+b\times Sq\left(y_{2}\right)$
Model 5	$a\times Sq\left(y_{1}\right)+b\times Sq\left(y_{2}\right)+c\times Sq\left(y_{1}\right)\times Sq\left(y_{2}\right)$
Model 6	$a\times Sq\left(y_{1}\right)+b\times Sq\left(y_{2}\right)+c\times Sq\left(y_{1}\times y_{2}\right)$

## Data Availability

The code that supports this article is available upon request from Shen Xu (xs.psych.pku@gmail.com) or at https://github.com/xspsych/review_code (accessed on 20 November 2022).
